# De-escalation of Axillary Surgery in Early-Stage Breast Cancer: A Comprehensive Narrative Review

**DOI:** 10.7759/cureus.105464

**Published:** 2026-03-18

**Authors:** Sweta Munagapati, Arnav Goyal

**Affiliations:** 1 General Surgery, Medical College of Georgia, Augusta University, Augusta, USA; 2 Internal Medicine, Wellstar Kennestone Regional Medical Center, Marietta, USA

**Keywords:** axilla, axillary lymph node dissection, axillary radiotherapy, breast cancer, de-escalation, lymphedema, neoadjuvant therapy, sentinel lymph node biopsy, targeted axillary dissection

## Abstract

Axillary management in early-stage breast cancer has shifted from routine axillary lymph node dissection (ALND) toward morbidity-sparing strategies that preserve oncologic outcomes. This narrative review summarizes the key practice-changing trials and contemporary guidance across the axillary de-escalation continuum: omission of completion ALND for limited sentinel lymph node metastasis, use of axillary radiotherapy when additional regional treatment is indicated, and omission of sentinel lymph node biopsy (SLNB) in carefully selected low-risk clinically node-negative patients. In the neoadjuvant setting, optimized SLNB and targeted axillary dissection can allow many initially node-positive patients to avoid ALND. Across scenarios, optimal axillary management increasingly depends on multidisciplinary integration of nodal burden, tumor biology, systemic therapy, and planned radiotherapy fields.

## Introduction and background

Axillary lymph nodes have long served as the primary regional marker of breast cancer spread [1]. For much of the 20th century, axillary lymph node dissection (ALND) was routinely performed, first within radical mastectomy and later within modified radical operations, with the goals of staging and regional control [1]. Nodal status remains prognostic and, in selected situations, still shapes recommendations for systemic therapy and radiotherapy [1].

The tradeoff of this approach is increased morbidity [2,3]. Lymphedema is the most recognized complication, but patients also experience seroma, numbness from intercostobrachial nerve injury, shoulder stiffness, and chronic pain that can persist for years [2,3]. These sequelae matter in a disease where long-term survival is common, making quality of life a major endpoint alongside oncologic control [2,3].

The introduction and validation of sentinel lymph node biopsy (SLNB) addressed much of this morbidity [2,4,5]. In clinically node-negative (cN0) disease, SLNB provides accurate staging with substantially less morbidity than ALND, and it has become the standard initial axillary operation for most patients with early-stage invasive breast cancer [2,4,5].

After SLNB became standard, completion ALND for limited sentinel node disease was challenged [6-8]. Randomized trials showed that many patients with one to two involved sentinel nodes, especially those treated with breast-conserving surgery (BCS) plus radiotherapy and systemic therapy, can omit completion ALND without sacrificing survival or regional control [6-8]. Other trials compared axillary radiotherapy with ALND and found excellent axillary control with lower lymphedema rates, supporting radiotherapy as an alternative when additional regional treatment is indicated [9-11]. More recent work has extended these concepts to selected patients undergoing mastectomy, where the combined plan for radiotherapy and the extent of nodal involvement guide decisions [12].

The field has moved further, asking whether SLNB can be avoided in some low-risk patients [13,14]. For many patients, adjuvant therapy decisions are driven by tumor subtype, genomic assays, and comorbidity, and nodal information may not change the plan [15-18]. Trials studying omission of SLNB in cN0 patients with negative axillary ultrasound (AUS) and favorable features report low axillary event rates and noninferior oncologic outcomes at early follow-up [13,14]. Guidelines now describe scenarios in which omission is reasonable, particularly for older patients with hormone receptor-positive, human epidermal growth factor receptor 2 (HER2)-negative disease when nodal results are unlikely to change management [15-18].

Neoadjuvant systemic therapy (NAST) adds another layer [19-22]. Nodal disease can respond completely to treatment, raising the question of whether ALND can be avoided even in patients who present with nodal involvement [19-22]. Prospective studies have defined technical steps that improve the accuracy of SLNB after neoadjuvant therapy, including dual-tracer mapping, retrieval of multiple sentinel nodes, and removal of a clipped node when feasible [19-22]. Regional nodal irradiation (RNI) has also expanded in selected higher risk groups and often intersects with surgical decisions, particularly when the goal is to control the axilla while limiting surgical morbidity [23-25].

Clinicians require a practical framework to apply this evidence: who should undergo SLNB, who can avoid completion ALND, when radiotherapy can substitute for surgery, and when ALND still has a role. This article summarizes the data across the de-escalation spectrum and highlights decision points that commonly arise in multidisciplinary breast cancer care.

## Review

This section synthesizes pivotal randomized trials, long-term follow-up studies, meta-analyses, and contemporary multidisciplinary guidance across the axillary de-escalation continuum in early-stage breast cancer, including upfront surgery, axillary radiotherapy as an alternative to ALND, omission of SLNB in selected low-risk patients, and neoadjuvant strategies such as targeted axillary dissection.

Approach to evidence selection

This is a narrative review intended to provide a practical synthesis of axillary de-escalation in early-stage breast cancer. Evidence was prioritized from landmark randomized trials, long-term follow-up publications, major multidisciplinary guidelines, and pivotal contemporary studies that inform common clinical decision points (upfront breast-conserving surgery, mastectomy, and NAST). The discussion is focused on practice-changing evidence and areas of ongoing controversy rather than an exhaustive technical description of operative technique.

Why de-escalate the axilla?

The rationale for axillary de-escalation rests on three principles [1]. First, in cN0 early breast cancer, axillary surgery often functions primarily as a staging tool rather than a curative intervention [1]. Second, contemporary systemic therapy reduces the risk of both distant and regional recurrence, decreasing the incremental benefit of extensive nodal surgery [1]. Third, ALND is associated with higher rates of lymphedema, paresthesias, seroma, pain, and shoulder dysfunction compared with SLN biopsy or observation; meta-analyses consistently show greater long-term lymphedema after more extensive axillary treatment [2,3].

Axillary anatomy, nodal staging, and pathologic terminology

Most de-escalation trials address low-burden disease in the low axilla (levels I-II), described anatomically relative to the pectoralis minor muscle [1]. Pathologic nodal burden is commonly categorized as isolated tumor cells (≤0.2 mm), micrometastases (>0.2-2.0 mm), and macrometastases (>2.0 mm); although these thresholds historically prompted completion ALND, contemporary management integrates the planned breast operation, radiotherapy fields, tumor biology, and systemic therapy [1,15].

Preoperative axillary evaluation

Clinical examination remains the first step in axillary staging [1]. For patients without palpable adenopathy, AUS is commonly used to assess nodal morphology, and suspicious nodes may be sampled with fine-needle aspiration or core biopsy [1]. In contemporary protocols evaluating SLN biopsy omission, a negative AUS is a key eligibility criterion [13,14,17,18]. Importantly, routine AUS-guided biopsy in otherwise low-risk cN0 patients can increase detection of minimal nodal disease and inadvertently escalate treatment; thus, imaging and biopsy strategies are best individualized within a multidisciplinary framework [15-18].

SLNB: technique and contemporary considerations

SLNB replaced ALND as the standard axillary staging procedure for clinically node-negative breast cancer because it provides accurate staging with substantially less morbidity [2,4,5]. For de-escalation decision-making, the most relevant technical considerations are reliable SLN identification (often supported by dual-tracer mapping) and adequate retrieval when the SLN result will alter radiotherapy planning or postneoadjuvant management (e.g., using dual tracer and retrieving ≥3 SLNs after neoadjuvant therapy) [19-22]. Because completion ALND is frequently omitted even when one to two SLNs contain metastasis, routine intraoperative frozen section is used less commonly when the result is unlikely to change immediate surgical management [6-8,15].

When is SLNB indicated?

Invasive Breast Cancer

For most patients with invasive breast cancer and a clinically negative axilla, SLN biopsy remains the preferred staging procedure when nodal information is expected to influence adjuvant management [1,15,16]. However, randomized trials and updated guidelines now support omission of SLN biopsy in narrowly defined low-risk groups treated with upfront breast-conserving therapy [13,14,17,18].

Ductal Carcinoma In Situ and Other In Situ Lesions

Because pure ductal carcinoma in situ lacks metastatic potential, SLN biopsy is generally not indicated for patients undergoing breast-conserving surgery [1,15,16]. SLN biopsy may be considered when mastectomy is planned (because mapping cannot be performed reliably afterward), when there is high suspicion of occult invasion, or when surgery may disrupt lymphatic drainage [1,15,16].

De-escalation after a positive sentinel node in upfront surgery

Historically, any SLN metastasis prompted completion ALND [1]. Multiple randomized controlled trials (RCTs) now demonstrate that, in selected patients with limited SLN disease, omitting completion ALND does not worsen survival or regional control [6-8,12]. Landmark and contemporary studies supporting these shifts are summarized in Table 1.

**Table 1:** Selected pivotal trials supporting omission of completion ALND and/or substitution with radiotherapy in patients with limited SLN disease ACOSOG: American College of Surgeons Oncology Group; ALND: axillary lymph node dissection; ART: adjuvant radiation therapy; BCS: breast-conserving surgery; DFS: disease-free survival; OS: overall survival; OTOASOR: Optimal Treatment Of the Axilla - Surgery Or Radiotherapy; RCT: randomized controlled trial; RT: radiation therapy; SLN: sentinel lymph node; SLNB: sentinel lymph node biopsy; WBRT: whole-breast radiotherapy

Trial	Population	Comparison	Local therapy context	Key oncologic outcomes	Morbidity signal
IBCSG 23-01 [6]	SLN micrometastases	No ALND vs. ALND	Mostly BCS; adjuvant therapy common	No DFS difference at 10 years	Less morbidity without ALND
ACOSOG Z0011 [7]	cT1-2, cN0; 1-2 positive SLNs	SLNB alone vs. ALND	BCS + WBRT; systemic therapy common	No OS difference; very low axillary recurrence	Less surgical morbidity with SLNB alone
AATRM [8]	SLN micrometastases	Observation vs. ALND	Mixed local therapy	No DFS difference	Fewer complications with observation
AMAROS [9,10]	Positive SLN	ART vs. ALND	BCS and mastectomy included	Equivalent axillary control	Lower lymphedema with ART
OTOASOR [11]	Positive SLN	Nodal RT vs. ALND	Single-center RCT	Similar DFS/OS; low regional recurrence	Supports RT as less morbid alternative
SENOMAC [12]	1-2 SLN macrometastases	Omit ALND vs. ALND	BCS and mastectomy; RT per protocol	Noninferior recurrence outcomes; very low axillary recurrence	Supports broader ALND omission with modern adjuvant therapy

Breast-Conserving Surgery With Whole-Breast Radiotherapy

The strongest evidence for omitting completion ALND applies to patients undergoing breast-conserving surgery (BCS) with whole-breast radiotherapy (WBRT) who have one to two positive SLNs [6-8]. In the American College of Surgeons Oncology Group (ACOSOG) Z0011, SLNB alone produced overall survival comparable to ALND, with low axillary recurrence [7]. International Breast Cancer Study Group 23-01 and AATRM further support omission of ALND for micrometastatic SLN disease [6,8]. Collectively, these trials support SLN biopsy alone as standard for many patients with limited nodal disease receiving contemporary systemic therapy and appropriate radiotherapy [1,6-8,15].

Radiotherapy Fields and “Z0011-Era” Practice

A longstanding question is whether the low axillary recurrence observed in Z0011 is partly attributable to incidental dose to level I-II nodes from tangential WBRT fields [1,18]. Dosimetric studies suggest that tangents, particularly “high tangents,” may partially cover the low axilla, but nodal coverage varies by technique and anatomy and is limited for level II-III nodes [1,18]. Accordingly, when Z0011 criteria are applied, radiotherapy planning should be explicit and coordinated across disciplines, especially for patients receiving partial-breast irradiation, prone-only techniques, or other nonstandard setups where incidental axillary dose may be minimal [1,18].

Mastectomy and the Question of ALND Omission

Earlier practice-changing data were most robust for BCS + WBRT, but more recent evidence supports ALND omission in selected mastectomy patients, particularly when postmastectomy RNI is planned [9,10,12]. AMAROS included mastectomy patients and demonstrated excellent axillary control with axillary radiotherapy compared with ALND, with less lymphedema [9,10]. SENOMAC enrolled both BCS and mastectomy patients and supports the omission of ALND for one to two SLN macrometastases when adjuvant therapy and radiotherapy are applied appropriately [12]. Importantly, contemporary analyses in biologically favorable, limited-node-positive disease treated with modern systemic therapy have reported very low locoregional recurrence rates even when comprehensive nodal irradiation is omitted, underscoring that the absolute benefit of routine postmastectomy radiation therapy (PMRT)/RNI may be small for carefully selected intermediate-risk patients and remains an active area of investigation [26]. When PMRT/RNI is not planned, the role of ALND omission is less certain and should be individualized based on estimated residual nodal burden and whether additional nodal information would change adjuvant therapy recommendations [1,15].

Axillary Radiotherapy as an Alternative to Completion ALND

When additional axillary treatment is desired beyond SLN biopsy, axillary radiotherapy offers an evidence-based alternative to ALND [9-11]. In AMAROS and Optimal Treatment Of the Axilla - Surgery Or Radiotherapy, nodal radiotherapy achieved regional control comparable to ALND while reducing lymphedema [9-11]. However, nodal irradiation can still contribute to lymphedema and shoulder stiffness, and optimal target volumes should be individualized according to baseline nodal risk, breast treatment, and the need for supraclavicular/internal mammary coverage [1,3,23-25].

Nodal Burden, Extranodal Extension, and Other Complex Clinical Factors

De-escalation decisions are most straightforward when trial eligibility criteria are met, but real-world practice often involves borderline features such as extranodal extension, multiple suspicious nodes on imaging, large tumors, or multifocal disease [14-18]. Professional society guidance generally supports ALND omission in Z0011-eligible patients and emphasizes multidisciplinary consideration when high-risk features accumulate [14-18]. In such cases, escalation may involve axillary radiotherapy or RNI rather than completion ALND, particularly when the goal is to reduce morbidity while maintaining regional control [9-11,23-25].

Omission of SLNB in selected cN0 patients

The next frontier in axillary de-escalation is the omission of SLN biopsy itself [13,14]. This approach is most attractive when nodal information is unlikely to change adjuvant management and when the baseline probability of nodal metastasis is low [13,14]. SOUND and Intergroup Sentinel Mamma provide the strongest randomized evidence supporting SLN biopsy omission in select cN0 populations (Table 2) [13,14]. Contemporary reviews synthesize these data and discuss the broader implications of SLNB omission strategies and ongoing trials [27].

**Table 2:** Selected trials evaluating omission of SLN biopsy in clinically node-negative early breast cancer INSEMA: Intergroup Sentinel Mamma; SOUND: Sentinel Node vs. Observation After Axillary Ultra-Sound; AUS: axillary ultrasound; BCS: breast-conserving surgery; SLNB: sentinel lymph node biopsy; RT: radiation therapy; QoL: quality of life

Trial	Key eligibility	Intervention	Comparator	Primary endpoint	Summary finding
SOUND [13]	cT1 (≤2 cm), cN0; negative AUS; mostly BCS	No axillary surgery	SLNB	Noninferior distant disease-free survival	No axillary surgery was noninferior to SLNB in selected patients
INSEMA [14]	cT1-2, cN0; BCS planned	Omit SLNB	SLNB	Noninferior invasive disease-free survival	Supports SLNB omission with improved arm outcomes
BOOG 2013-08 (ongoing) [28]	cT1-2, cN0; BCS + RT	Omit SLNB	SLNB	Distant metastasis-free survival (planned)	Primary oncologic results pending; QoL benefits anticipated

Patient selection and guideline recommendations

ASCO's 2025 guideline update endorses the omission of SLN biopsy for a narrowly defined low-risk group treated with upfront breast-conserving therapy [17,18]. Recommended criteria include postmenopausal patients aged ≥50 years with grade 1-2, hormone receptor-positive, HER2-negative tumors ≤2 cm, a clinically node-negative axilla confirmed by ultrasound, and a plan for WBRT and endocrine therapy [17]. Outside these criteria, SLN biopsy remains standard because nodal information may influence decisions about RNI and systemic therapy [1,15-18].

When Does Nodal Staging Still Change Management?

Even as omission strategies expand, SLN biopsy remains valuable when nodal status is likely to alter adjuvant recommendations [1,15-18]. Examples include decisions regarding RNI after BCS, selection of postmastectomy radiation fields, and adjuvant systemic therapy escalation in biologically aggressive subtypes or when genomic risk tools are unavailable or equivocal [1,15-18,23-25]. Conversely, when systemic therapy is planned regardless of nodal status (or when comorbidity limits escalation) and when radiotherapy plans are fixed, the incremental value of SLN biopsy decreases [1,15-18].

Older Patients and Choosing Wisely Recommendations

The Society of Surgical Oncology's Choosing Wisely recommendations discourage routine SLN biopsy in women aged ≥70 years with clinically node-negative, early-stage, hormone receptor-positive, HER2-negative breast cancer when results are unlikely to alter management [16]. This aligns with the principle that the clinical value of nodal staging decreases when systemic therapy decisions are driven predominantly by tumor biology, comorbidity, and patient preference [1,15-18].

NAST and axillary de-escalation

Clinically Node-Negative Patients Receiving NAST

For patients who are cN0 at presentation but receive NAST, SLN biopsy after therapy is commonly used to provide pathologic staging while avoiding ALND [1]. In many cases, nodal status after NAST influences decisions about RNI and adjuvant therapy escalation [1]. Routine omission of SLN biopsy after NAST remains investigational [1].

Initially Node-Positive Patients Converted to ycN0

In patients presenting with biopsy-proven node-positive disease, NAST can eradicate nodal metastases in a meaningful subset, particularly in HER2-positive and triple-negative subtypes [19-22]. ACOSOG Z1071, SENTinel NeoAdjuvant, and Sentinel Node Biopsy Following Neoadjuvant Chemotherapy evaluated SLN biopsy after NAST and highlighted that false-negative rates are unacceptably high when few nodes are retrieved, but improve with dual-tracer mapping and retrieval of greater than or equal to three SLNs [19,20,22]. Targeted axillary dissection (TAD), defined as SLN biopsy plus removal of the biopsy-proven clipped node, further improves accuracy and is increasingly used to spare patients from ALND after nodal response (Table 3) [21].

**Table 3:** Key postneoadjuvant axillary staging studies and the rationale for targeted axillary dissection SN FNAC: Sentinel Node Biopsy Following Neoadjuvant Chemotherapy; SLNB: sentinel lymph node biopsy; NAST: neoadjuvant systemic therapy; ACOSOG: American College of Surgeons Oncology Group; ALND: axillary lymph node dissection; SLN: sentinel lymph node; TAD: targeted axillary dissection; SENTINA: SENTinel NeoAdjuvant

Study	Population	Strategy	Key message
SN FNAC [19]	Biopsy-proven node-positive	SLNB after NAST with standardized pathology	Supports feasibility with optimized technique
ACOSOG Z1071 [20]	Biopsy-proven cN1-2; post-NAST staging	SLNB then ALND	Technique (dual tracer; ≥3 SLNs) lowers false-negative rates
TAD [21]	cN+ with clipped node	SLNB + clipped node excision	Improves accuracy compared with SLNB alone
SENTINA [22]	cN+ → ycN0 cohort included	SLNB after NAST	False-negative rate improves with higher SLN yield

Residual Nodal Disease After NAST: Can ALND Be Avoided?

When residual nodal disease persists after NAST (ypN+), completion ALND remains common practice [1]. However, ongoing randomized trials such as Alliance A011202 are evaluating whether axillary and regional nodal radiotherapy can replace ALND in patients with residual nodal metastases after NAST [29].

RNI and its interaction with surgical de-escalation

RNI can reduce regional recurrence and modestly improve disease-free survival in selected populations [23-25]. Randomized trials including MA.20 and EORTC 22922/10925 support RNI in patients with node-positive disease and in some high-risk node-negative patients [23-25]. In de-escalation paradigms, RNI and axillary radiotherapy can substitute for completion ALND or provide coverage when nodal surgery is minimized, but the incremental lymphedema risk of adding nodal irradiation should be considered [3].

Situations where ALND remains appropriate

Despite broad de-escalation, ALND remains an important tool for selected patients in whom it is expected to improve regional control or provide critical staging information [1,15]. Across contemporary guidelines, common indications include 1) clinically node-positive axilla with bulky/matted disease not adequately addressed by SLN-based strategies, 2) persistent clinically positive nodes after NAST when less invasive staging is unreliable or incomplete, 3) inflammatory breast cancer, and 4) situations in which accurate nodal burden quantification is essential for adjuvant therapy planning and cannot be obtained otherwise [1,15].

In addition, completion ALND may be considered when patients clearly fall outside the eligibility of major de-escalation trials and are not candidates for axillary radiotherapy or RNI (e.g., inability to receive radiotherapy, extensive nodal involvement, or surgeon concern for uncontrolled regional disease) [1,15-18]. These decisions are best made in a multidisciplinary setting with explicit discussion of tradeoffs between oncologic certainty and morbidity [1,15-18].

Special clinical scenarios

Local Recurrence and Prior Axillary Surgery

Patients presenting with ipsilateral breast tumor recurrence after prior SLN biopsy or ALND represent a distinct challenge [1,15]. Reoperative SLN biopsy is feasible in selected patients, but lymphatic drainage can be altered and mapping success varies; when mapping is unsuccessful or there is overt nodal disease, management is individualized and often guided by imaging, prior radiotherapy fields, and planned systemic therapy [1,15].

Pregnancy

In pregnancy-associated breast cancer, axillary staging should balance maternal oncologic needs with fetal safety [1]. Most guidelines support SLN biopsy with a technetium-99m radiotracer when indicated, while use of blue dye is often avoided because of rare anaphylaxis risk and limited pregnancy data; institutional practice varies, and multidisciplinary coordination is essential [1].

Male Breast Cancer and Other Uncommon Histologies

Although high-level de-escalation trials primarily enrolled women, the principles of axillary staging and morbidity reduction are generally extrapolated to male breast cancer and uncommon histologies [1]. When systemic therapy and radiotherapy plans are analogous, SLN-based strategies are typically favored over ALND in clinically node-negative disease [1].

Morbidity, patient-reported outcomes, and survivorship

The extent of axillary treatment is a major determinant of long-term complications [2,3]. In National Surgical Adjuvant Breast and Bowel Project B-32 and other studies, SLN biopsy was associated with less morbidity than ALND [2,4]. Meta-analyses estimate substantial lymphedema risk after ALND, particularly when combined with nodal irradiation [1,3]. Consistent with this, AMAROS reported lower lymphedema rates with axillary radiotherapy compared with ALND [9,10], and SLN-omission trials report improved arm symptoms and function in patients who avoid axillary surgery [13,14].

Risk reduction when ALND is unavoidable

ALND remains necessary for some patients (e.g., persistent clinically positive axilla after NAST, bulky nodal disease, inflammatory breast cancer, or extensive nodal involvement) [1,15]. For these patients, morbidity-mitigation strategies may include structured postoperative physical therapy and prospective limb surveillance [1]. Specialized techniques such as axillary reverse mapping (ARM) and immediate lymphatic reconstruction (also called LYMPHA or immediate lymphatic reconstruction) may reduce lymphedema risk when performed by experienced teams [30-32].

Practical approach to axillary de-escalation

Contemporary axillary management can be summarized into common clinical pathways (Table 4) [1,15-18]. The overarching principle is that axillary treatment intensity should match the expected clinical value of nodal information and baseline risk of residual nodal disease, while accounting for planned radiotherapy and systemic therapy [1,15-18].

**Table 4:** Pragmatic axillary management pathways in the de-escalation era ALND: axillary lymph node dissection; ART: adjuvant radiation therapy; AUS: axillary ultrasound; BCS: breast-conserving surgery; DCIS: ductal carcinoma in situ; NAST: neoadjuvant systemic therapy; PMRT: postmastectomy radiation therapy; RNI: regional nodal irradiation; RT: radiation therapy; SLN: sentinel lymph node; SLNB: sentinel lymph node biopsy; TAD: targeted axillary dissection; WBRT: whole-breast radiotherapy

Scenario	Typical axillary strategy	When to escalate	Key notes
Upfront BCS + WBRT, cN0	SLNB; or omit SLNB in ASCO-defined low-risk group [17]	Suspicious nodes on exam/AUS or if nodal status will alter adjuvant plan	Omission requires negative AUS and multidisciplinary agreement [17,18]
Upfront BCS + WBRT, 1-2 positive SLNs	Omit completion ALND (Z0011/IBCSG criteria) [6-8]	≥3 positive SLNs, gross nodal disease, or inability to deliver appropriate RT	Consider ART/RNI based on risk and radiation plan [9-11,23-25]
Upfront mastectomy, cN0	SLNB (standard) [1,15]	If SLN positive and PMRT/RNI not planned, consider ALND vs. ART	Evidence supports ALND omission when RNI is planned in selected patients [9,10,12]
NAST for biopsy-proven cN+ → ycN0	TAD or optimized SLNB [19-22]	Persistent clinically positive nodes or inadequate node retrieval	Aim for dual tracer and retrieval of ≥3 SLNs when feasible [19,20,22]
NAST with residual nodal disease (ypN+)	ALND (common standard) ± RNI	Clinical trial enrollment when available	Alliance A011202 may redefine standard [29]
Pure DCIS treated with lumpectomy	No SLNB [16]	If mastectomy planned or high suspicion of invasion	Discuss SLNB at mastectomy because staging later is unreliable [1,15]

Ongoing trials and future directions

Ongoing RCTs will further define minimal effective axillary treatment. POsitive Sentinel NOde: adjuvant therapy alone versus adjuvant therapy plus Clearance or axillary radiotherapy is evaluating whether any additional axillary treatment (ALND or axillary radiotherapy) can be omitted in patients with limited SLN macrometastasis receiving modern systemic therapy [33]. TAXIS investigates tailored axillary surgery in clinically node-positive disease, aiming to reduce morbidity compared with ALND while preserving locoregional control [34]. Long-term follow-up of SLN-omission trials and future integration of advanced imaging, response-adapted therapy, and molecular prognostic tools may enable further reduction of axillary interventions [13,14,17,18].

Limitations

This review is narrative in design and does not follow formal systematic review methodology or Preferred Reporting Items for Systematic reviews and Meta-Analyses guidelines. Evidence was selected to highlight landmark randomized trials, guideline-defining studies, and commonly encountered clinical decision points, and therefore is not intended to be exhaustive. Quantitative synthesis was not undertaken. Additionally, long-term follow-up remains limited for several SLNB-omission and postneoadjuvant de-escalation strategies, and optimal integration of radiotherapy fields with minimized surgery continues to evolve [13,14,17,18].

## Conclusions

Contemporary axillary management aims to minimize morbidity without compromising regional control or survival by tailoring axillary intervention to nodal burden, tumor biology, systemic therapy, and planned radiotherapy fields. For most Z0011-eligible patients undergoing breast-conserving surgery with whole-breast radiotherapy, completion ALND can be safely omitted when one to two SLNs are positive, with axillary radiotherapy or RNI reserved for selected higher risk scenarios. Randomized evidence and recent guideline updates now support omitting SLNB itself in carefully selected low-risk clinically node-negative patients with negative AUS when nodal information is unlikely to alter adjuvant management. After NAST, optimized SLNB and TAD can reduce ALND use in initially node-positive patients who downstage, but standardization and long-term outcomes remain important areas for ongoing study. Key unresolved questions include refining which intermediate-risk mastectomy patients derive meaningful benefit from PMRT/RNI in the modern systemic era and how best to incorporate imaging, biology, and treatment response into unified de-escalation pathways.
